# A 3-Year Longitudinal Study of Effects of Parental Feeding Practices on Child Weight Status: The Childhood Obesity Study in China Mega-Cities

**DOI:** 10.3390/nu14142797

**Published:** 2022-07-07

**Authors:** Lu Ma, Na Yan, Zumin Shi, Yixin Ding, Siran He, Zhengqi Tan, Bo Xue, Yating Yan, Cai Zhao, Youfa Wang

**Affiliations:** 1 Global Health Institute, School of Public Health, Xi’an Jiaotong University Health Science Center, Xi’an 710061, China; malu1990@xjtu.edu.cn (L.M.); yanna19960421@gmail.com (N.Y.); dingyx226@stu.xjtu.edu.cn (Y.D.); 3121115097@stu.xjtu.edu.cn (Y.Y.); zhaocai@stu.xjtu.edu.cn (C.Z.); 2 Human Nutrition Department, College of Health Sciences, QU Health, Qatar University, Doha 2713, Qatar; zumin.shi@gmail.com; 3 Milken Institute School of Public Health, The George Washington University, Washington, DC 20007, USA; siranhe@gmail.com; 4 Department of Biostatistics and Epidemiology, University of North Texas Health Science Center, Fort Worth, TX 76107, USA; zqtansh@gmail.com; 5 Institute of Nutrition and Food Safety Risk Monitoring, Shaanxi Center for Disease Control and Prevention, Xi’an 710061, China; liying2021a@stu.xjtu.edu.cn

**Keywords:** general obesity, central obesity, parental feeding practices, children, China

## Abstract

This study examined the longitudinal associations between parental feeding practices and child weight status, and their potential modification effects by child sex, age, and maternal and paternal educations among children. Data were collected from 2015 to 2017 of 2139 children aged 6–17 years and their parents in five Chinese mega-cities. Parental feeding practices were assessed using 11-items from Child Feeding Questionnaire. Waist-to-height ratio (WHtR), body mass index (BMI), and general and central obesity were measured and analyzed using a mixed-effects model. Three parental feeding patterns were identified by factor analysis including “concern”, “pressure to eat”, and “control”. Concern was associated with higher BMI z-score, WHtR (βs ranged from 0.01 to 0.16), and general obesity (ORs ranged from 1.29 to 6.41) among children aged ≤12 years and >12 years, regardless of child sex and parental educations. Pressure to eat was associated with lower BMI z-score (β = −0.08, *p* < 0.001), WHtR (β = −0.004, *p* < 0.01), and general (OR = 0.53, 95% CI = 0.42, 0.66) and central obesity (OR = 0.72, 95% CI = 0.58, 0.90) among children aged ≤12 years. Further analyses showed that significant associations were found for children with maternal or paternal education of college and above. Control was associated with increased risk of general and central obesity among children with maternal education of college and above, regardless of age. Our study indicates that higher concern and lower pressure to eat were associated with increased risk of obesity among children. Control was associated with increased risk of obesity among children with maternal education of college and above. Future childhood obesity preventions may optimize parental feeding practices.

## 1. Introduction

Obesity has become a serious public health problem worldwide [1,2]. In China, national data show that about 20% of school-age children had overweight and obesity in 2020; it was projected that the prevalence would continue to increase in coming years, especially in mega-cities [3]. Childhood obesity leads to many adverse health outcomes, such as hypertension and diabetes [4]. Additionally, the persistence of childhood obesity into adulthood and its attributable morbidity is a major health problem [5]. It is crucial to identify and intervene modifiable determinants of obesity early in life [6].

Evidence indicates that parental beliefs, attitudes, and feeding practices may influence childhood obesity through affecting child eating behaviors and nutritional intake [7]. Parents, as major food providers to their children, enforcers of eating behaviors, and role models in dietary intake, can have a strong impact on shaping their children and adolescents’ weight status [8,9]. A 2015 systematic review of 21 studies in children aged 4- to 12-years showed that specific feeding practices such as restriction and pressure to eat were associated with body mass index (BMI) in most cross-sectional studies [10]. Only two longitudinal studies were included in this systematic review, which reported an association between parental feeding practices and weight status [11,12]. Another longitudinal study among 74 white children aged 11.0 years found that parental feeding practices were not associated with total fat mass [13]. However, these studies were conducted in European and North American countries; to our knowledge, no such longitudinal studies have been conducted in China [14].

Previous studies targeted children before puberty [15,16]; however, the independence and eating behaviors of younger and older children may be different [17]. Nonetheless, the associations between parental feeding practices and obesity risk among children after puberty remain unclear. Moreover, the Asian population has a lower BMI, but has higher total and central adiposity for a given body weight when compared with matched white population, which makes them more susceptible to metabolic diseases. Therefore, in addition to BMI, central obesity-related indicators should be measured for Chinese children [18]. However, no study has examined the associations between parental feeding practices and the risk of central obesity among children. Therefore, longitudinal evidence on the associations of parental feeding practices with both general and central obesity risks among children are needed.

Furthermore, studies have indicated that parental feeding practices are influenced by children’s biological sex, as well as maternal and paternal educations. In east Asia, parents tend to place greater emphasis on managing the weight and eating behaviors of girls rather than boys, which may be due to societal standards of female body image [19]. Parental educations have been found to be associated with their ability to process health information, leading to improved health-related decisions for their children [20]. Though child sex and parental educations have been established to play a role in parental feeding practices, few studies have examined the influence of such factors on parental feeding practices about childhood obesity [5,8].

This study aimed to examine: (1) longitudinal associations between parental feeding practices and child weight status including BMI, waist-to-height ratio (WHtR), general obesity, and central obesity; and (2) whether such associations are modified by child sex, child age, and maternal and paternal educations by utilizing three-year longitudinal data collected from five mega-cities across China in 2015, 2016, and 2017. We hypothesized that parental feeding practices would be longitudinal associated with child weight status, and such associations would be stronger among girls, younger children, and children with parents having higher educations.

## 2. Materials and Methods

### 2.1. Study Design and Participants

The Childhood Obesity Study in China Mega-Cities (COCM) was a longitudinal study aiming to examine the etiology of childhood obesity and chronic diseases in China [21,22]. This study uniquely captures health trends related to lifestyle behavioral changes occurring at the forefront of China’s economic growth. Four major cities were included at baseline in 2015, including Beijing (China’s capital, in the north), Shanghai (the largest city in the east), Nanjing (China’s old capital, in the east), and Xi’an (the largest city in the west). The COCM baseline data was collected in 2015 and was followed up in 2016 and 2017. In 2016, a fifth city, Chengdu (the largest city in the southwest), was added to the study [23,24]. Each city has a population size greater than 8 million.

In each city, two primary schools and two middle schools were randomly selected. In each school, a class from each grade (grades 3–6 in primary schools and grades 7–8 in middle schools) was then randomly selected. In total, 48 classes from 8 primary schools and 8 middle schools were included at baseline in 2015. In 2016 and 2017, 12 classes including 8 classes from 2 primary schools and 4 classes from 2 middle schools were added in Chengdu. Therefore, 60 classes from 20 schools were included in 2016 and 2017.

The current study used data collected in 2015, 2016, and 2017 on child and parental sociodemographic characteristics, parental feeding practices, and child body weight status. Child sociodemographic characteristics and eating behaviors were self-reported by children under the guidance of professionals in classes in schools. Child weight status indicators were objectively measured by professionals in schools based on standard protocol. The children’s mothers (or other primary care givers if mothers were absent) self-reported their socio-demographic characteristics, family characteristics, parental feeding practices and attitudes, and anthropometrics at home. A total of 2139 parent-child dyads with parental feeding practices and child anthropometric measurements having been recorded at least twice from 2015 to 2017 were included in the longitudinal data analyses.

### 2.2. Key Variables and Measurements

#### 2.2.1. Outcome Variables

General obesity: The participants’ BMI was calculated as weight (in kilograms) divided by squared height (in meter) squared. Height was measured using Seca 213 Portable Stadiometer Height-Rods (Seca China, Hangzhou, China) in duplicates with a precision of 0.1 cm. Body weight was measured using Seca 877 electronic flat scales (Seca China, Hangzhou, China) in duplicates with a precision of 0.1 kg. Height and weight were measured by trained health professionals. General obesity was defined using sex- and age-specific BMI cutoff points according to the Chinese National Standard, “WS/T 586-2018 Screening for overweight and obesity among school-aged children and adolescents” (underweight/normal weight: < 85th percentile; 85th percentile ≤ overweight < 95th percentile; 95th percentile ≤ obesity) [25]. In this study, general obesity included overweight and obesity.

Central obesity: Waist circumference was measured using a non-stretchable tape in duplicates with a precision of 0.1 cm. WHtR was calculated as waist circumference (m) divided by height (m). Central obesity was defined as having a WHtR ≥ 0.48 [26].

#### 2.2.2. Exposure Variables

Parental feeding practices: Parental attitude and practices towards child feeding and obesity proneness were assessed by an 11-item scale adapted from the Child Feeding Questionnaire (CFQ) [27,28]. CFQ presents good validity and reliability among Chinese children [29] and adolescents [30]. Children’s primary caregivers were asked to rate whether they agree with the statements using a 5-point Likert scale where “Strongly disagree” = 1 and “Strongly agree” = 5. Three factors were identified and labeled as “Concern”, “Pressure to eat”, and “Control” from factor analysis in this study (Table 1). “Concern” assessed parental concerns about their child’s risk of being overweight; “Pressure to eat” assessed the extent to which parents put certain pressure on their children to modify eating behaviors; and “Control” assessed the extent to which parents actively control what their children eat. Total score was calculated for each factor with higher scores indicating higher engagement in particular child feeding practices. The Cronbach’s alpha for the overall scale was 0.65, and the Cronbach’s alpha for “Concern”, “Pressure to eat”, and “Control” were 0.66, 0.56, and 0.32, respectively. Each score of the three factors were also recorded into tertiles.

#### 2.2.3. Covariates

Both child and parental characteristics were included as covariates. These variables included: age (years), sex (boy or girl), city of residence (Beijing, Shanghai, Nanjing, Xi’an, or Chengdu), survey years, child school level (primary or secondary), maternal and paternal BMI and educations (middle school or lower, high or vocational school, or college or above). Child age group (≤12 years and >12 years) and sex, and maternal and paternal educations were also used as potential modifying factors in the stratified analyses. Characteristics of parental feeding practices across these modifying factors were shown in Supplemental Table S1.

### 2.3. Statistical Analysis

Chi-square test (for categorical variables) and ANOVA (for continuous variables) were conducted to examine the baseline differences of child and parental characteristics across parental feeding practices tertiles.

Exploratory factor analysis (EFA) was conducted to identify sets of items among parental feeding practices that reflect underlying factors of these practices. Kaiser–Meyer–Olkin (KMO) Measure of Sampling Adequacy and Bartlett’s Test of Sphericity were used to assess the suitability of the respondent data for factor analysis, and principal-component factor was used to extract factors and three factors were retained, and varimax rotation was used. The number of factors to retain was determined by eigenvalues >1 indicating a factor that explains more variance than any individual item of parental feeding practice [31,32]. Chi-square test was conducted to compare the prevalence of general and central obesity across the tertiles of each identified parental feeding practices pattern.

Due to the within-child variability in parental feeding practices and weight status indicators (Supplemental Table S2), a three-level (cities, schools, and individuals) longitudinal mixed-effects model was used to examine: (1) associations between parental feeding practices and weight status indicators, with linear models for BMI z-score and WHtR (continuous variables) as dependent variables, and logistic models for general and central obesity (binary variables) as dependent variables; (2) whether associations between parental feeding practices and child weight status were modified by child sex and maternal and paternal educations; and (3) child sex- and maternal and paternal education-stratified analyses of such associations. The final models adjusted for random effects arising from child city of residence, child school level, as well as other covariates. Separate models were tested for each parental feeding practice. In the child sex- and maternal and paternal educations-stratified analyses, all covariates were adjusted except for the stratifying variable.

Effect sizes were presented as beta coefficients for continuous outcomes and odds ratios (ORs) for categorical variables with a 95% confidence interval (CI). Analyses were performed using Stata 15 (StataCorp, College Station, TX, USA). Statistical significance was set at *p* < 0.05 (two-sided).

## 3. Results

### 3.1. Demographic Characteristics and Health Outcomes across Parental Feeding Practice Tertiles

The mean age for children ≤ 12 years was 9.82 ± 1.34 years, and for children >12 years was 13.52 ± 1.59 years. From lower to higher tertile of concern score, the prevalence of general obesity and central obesity increased (all *p*-values < 0.001). The prevalence of general obesity but not central obesity decreased across tertiles of pressure to eat (*p* = 0.001). However, no significant differences were found in the prevalence of general obesity and central obesity across control tertiles (Table 2).

### 3.2. Child Age- and Sex-, and Parental Educations-Stratified Longitudinal Associations between Concern and Child Weight Status

**Figure 1:**
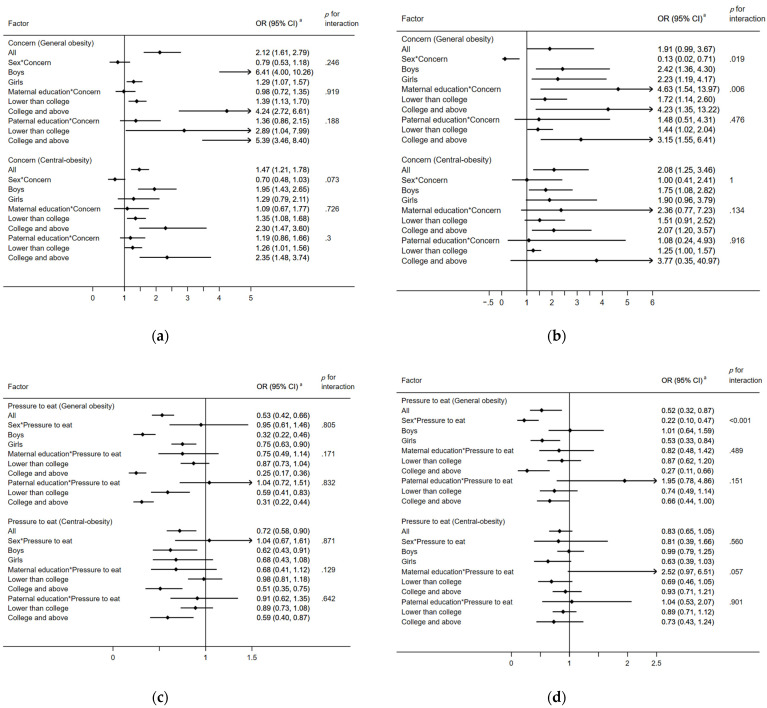

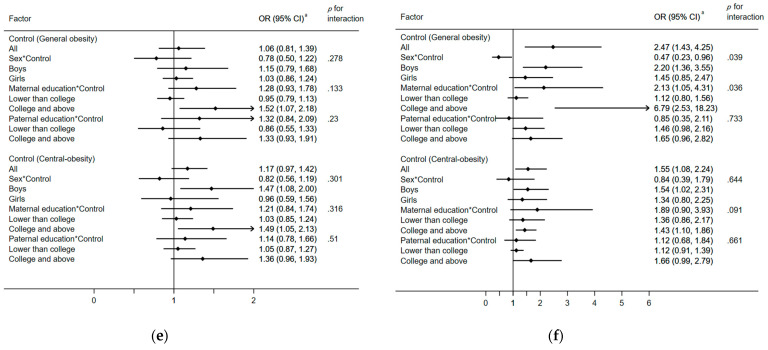
Longitudinal associations of parental feeding practices with general obesity and central obesity of children, stratified by child age, child sex, and maternal and paternal educations—the Childhood Obesity Study in China Mega-Cities (*n* = 2139). (**a**) Concern—children (≤12 years); (**b**) Concern—children (>12 years); (**c**) Pressure to eat—children (≤12 years); (**d**) Pressure to eat—children (>12 years); (**e**) Control—children (≤12 years); (**f**) Control—children (>12 years). ^a^: Child sex, child school level, child general obesity (or central obesity), child city of residence, maternal and paternal BMI, and maternal and paternal educations were adjusted as covariates in the mixed-effects model. In child age- and sex-stratified and maternal and paternal education-stratified analyses, models adjusted for the same variables except for child age and child sex, and maternal and paternal educations. Analysis of each parental feeding practice was conducted in 28 separate mixed-effects models for one specific outcome. *: Means the two variables were multiplied and added as an interaction term in the mixed-effects model. Variable definition: General obesity was defined using sex- and age-specific BMI cutoff points according to the Chinese National Standard, “WS/T 586-2018 Screening for overweight and obesity among school-aged children and adolescents”. In this study, general obesity included overweight and obesity. Central obesity was defined as having a WHtR ≥ 0.48. A higher score is indicative of higher engagement in particular child feeding practices.

Among all children aged ≤ 12 years, concern was consistently associated with higher BMI z-score (β = 0.13, 95% CI = 0.09, 0.17), WHtR (β = 0.01, 95% CI = 0.005, 0.01), and the risks of general obesity (OR = 2.12, 95% CI = 1.61, 2.79) and central obesity (OR = 1.47, 95% CI = 1.21, 1.78). Similar such associations were found for children aged > 12 years. For general obesity, concern was consistently associated with higher BMI z-score, WHtR, and the risk of general obesity, regardless of child sex- and maternal- and paternal- educations. Concern was associated with increased risk of central obesity, regardless of maternal- and paternal-educations; however, such associations were only significant for boys (Table 3 and Figure 1).

Among all children aged > 12 years, concern was consistently associated with higher BMI z-score, WHtR, and the risk of general obesity, regardless of child sex and maternal and paternal educations. Child sex and maternal education modified such associations. Concern was associated with the risk of central obesity only among boys and children with mothers having college or above education (Table 3 and Figure 1).

### 3.3. Child Age- and Sex-, and Parental Educations-Stratified Longitudinal Associations between Pressure to Eat and Child Weight Status

Among all children aged ≤12 years, pressure to eat was associated with lower BMI z-score (β = −0.08, 95% CI = −0.12, −0.04), WHtR (β = −0.004, 95% CI = −0.01, −0.001), and the risks of general obesity (OR = 0.53, 95% CI = 0.42, 0.66) and central obesity (OR = 0.72, 95% CI = 0.58, 0.90). In general, child sex did not modify such associations. Maternal and paternal educations modified such associations, and pressure to eat was associated with lower BMI z-score, WHtR, and the risk of general obesity and central obesity among children with maternal or paternal education of college or above (Table 3 and Figure 1).

Among all children aged >12 years, pressure to eat was not associated with BMI z-score, WHtR, and the risk of general obesity and central obesity, regardless of maternal and paternal educations. Child sex modified such associations; pressure to eat was associated with lower BMI z-score (β = −0.08, 95% CI = −0.14, −0.01), WHtR (β = −0.005, 95% CI = −0.01, −0.001), and the risk of general obesity (OR = 0.53, 95% CI = 0.33, 0.84) among girls, but not boys (Table 3 and Figure 1).

### 3.4. Child Age- and Sex-, and Parental Educations-Stratified Longitudinal Associations between Control and Child Weight Status

Among all children aged ≤12 years, control was not associated with any of the weight status indicators, regardless of child sex and paternal educations. However, maternal education modified such associations; control was associated with higher risk of general obesity (OR = 1.52, 95% CI = 1.07, 2.18) and central obesity (OR = 1.49, 95% CI = 1.05, 2.13) among children with mothers having college or above education (Table 3 and Figure 1).

Among all children aged >12 years, control was also not associated with any of the weight status indicators, regardless of child sex and paternal educations. Maternal education also modified such associations; control was associated with higher risk of general obesity (OR = 6.79, 95 %CI = 2.53, 18.23) and central obesity (OR = 1.43, 95 %CI = 1.10, 1.86) among children with mothers having college or above education (Table 3 and Figure 1).

## 4. Discussion

The present longitudinal study demonstrated that parental feeding practices predicted increased childhood obesity risks, and child sex, child age, and parental educations modified such associations. The concern pattern was a consistent risk factor of general and central obesity among children aged ≤ 12 years, regardless of child sex, maternal and paternal educations. An exception was that concern was not associated with the risk of central obesity in girls aged ≤ 12 years. Pressure to eat was a protective factor of general and central obesity only among children aged ≤ 12 years. Further analyses found that such statistically significant associations between pressure to eat and obesity risk only presented among younger children with maternal or paternal education of college or above. Control was only associated with higher risks of general obesity and central obesity among children ≤ 12 years and > 12 years with maternal education of college or above.

Consistent with the findings of several other cross-sectional studies among children aged ≤ 12 years [8,9], our results further provided the longitudinal evidence that parental concern feeding pattern was a risk factor of obesity in children ≤ 12 years. Moreover, our findings added to the current literature that parental concern feeding pattern was a risk factor of general obesity among children aged > 12 years, regardless of child sex and maternal and paternal educations. In the CFQ, concern measures parental concerns about their children’s risk of being overweight. Studies have shown that parental concern about child weight status rarely translated into healthier feeding practices or family meal characteristics [33,34]. Conversely, parents who are concerned about their child’s weight reported more negative parental practices (i.e., pressure to eat) and less health-promoting parental practices (i.e., parents join the children to exercise) [33,34]. Consistent with these findings, we found that the frequency of eating out was high in the third tertile of concern compared with the first tertile of concern (Supplemental Table S4). These negative parental feeding practices have detrimental effects on children’s relationships with food and self-regulation of eating, which could lead to excess weight gain [33].

In this study, about 55% of parents reported concerns about their children’s weight status. Future childhood obesity interventions targeting family contextual factors may be considered to help parents understand and be responsive to children’s hunger and satiety cues [7] and provide families with practical strategies for healthy eating promotion, rather than heighten parents’ concerns about their children’s weight status.

Innovatively, we found that the associations between pressure to eat and childhood obesity were modified by child age. Though pressure to eat predicted lower risks of general and central obesity in children ≤ 12 years, we added to the current literature that it was not associated with general nor central obesity among children aged > 12 years. Previous cross-sectional studies [35,36] and longitudinal studies [8,37] reported that pressure to eat was related to lower risk of obesity among younger children. Pressure to eat may disrupt younger children’s development of self-regulated eating, decrease food enjoyment and actual consumption of provided food, and increase food avoidance [38] and reliance on external cues when eating [39]. Children aged > 12 years enter the life stage of adolescence in which individuals may start to seek autonomy in their behaviors and food choice [40]. Thus, adolescents may be less influenced by parental pressure to eat, therefore, pressure to eat was not associated with obesity risk.

The parental educations-stratified analyses further revealed that associations between pressure to eat and younger children’s weight status were modified by maternal and paternal educations. When parents urge their children to increase food intake, only children with maternal or paternal education of college or higher tend to subsequently lose weight over time. This may be because parents with high education may have higher health literacy [41], which may be associated with healthier eating habits in children [42] and decreased risk of childhood obesity [43]. Moreover, other factors that often tightly relate to education (e.g., nature of employment, income) may also affect eating behaviors of children and their weight status. For example, a study suggested that efforts to promote formalized employment among mothers may be an effective method for improving diet diversity and feeding frequency in low- and middle-income countries [44].

We did not find previous studies examining the potential modifying effect of maternal and paternal educations on the longitudinal associations between pressure to eat and child weight status. One study by Ayine et al. found that pressure to eat indirectly predicted BMI z-score through maternal education in children aged 6–10 years [9]. In our study, parents with education of college or above were more likely to pressure children to eat (Supplemental Table S1). In contrast, in Ayine et al. study, maternal education was inversely related to pressure to eat in the U.S. [9], indicating that the modifying effect of paternal and maternal educations on the associations between pressure to eat and childhood obesity requires careful interpretation among different study populations.

In the present study, control pattern was associated with increased risk of general and central obesity among both children ≤ 12 years and > 12 years. These findings may be potentially due to the fact that controlling a child’s food intake may limit his/her ability to self-regulate food consumption and to properly identify and react to hunger and satiety cues, which may be potentially associated with obesity [45,46]. Further stratified analyses found that the effect of control pattern on childhood obesity only for children whose maternal education was college or above. We compared the control feeding practices of mothers with education lower than college and college or above (Supplemental Table S1) and found that mothers with a college education or above were more likely to “offer snacks to the child as a reward for good behavior”. This feeding practice may contribute to the development of unhealthy eating habits [47] (emotional eating and fussiness), which may lead to obesity.

This study has several strengths. First, we objectively measured both general and central obesity outcomes, and investigated the three-year longitudinal associations between parental feeding practices and these outcomes. Second, uniquely, besides among children aged ≤ 12 years, we added to the current literature on such associations among children aged > 12 years. Third, sex- and maternal and paternal educations-stratified analyses were conducted to provide more clear longitudinal associations between parental feeding practices and child weight status.

This study has limitations. First, we used 11 items from the validated CFQ to assess parental feeding practices [29], but not the full CFQ. Although the modified CFQ presents acceptable reliability, it has yet to be validated in Chinese adolescents. Moreover, the primary caregiver’s self-reported measures of parental feeding practices may result in social-desirability bias or possible misclassification. Future studies could complement such self-report measures by adding observation measures [48]. Second, the reliability of the factor “control” was relatively low. This may be because only three items were included in this construct. We assessed associations between each item in this factor and child weight status, the results for all children and parental educations- and sex-stratified were similar to that of the whole factor (Supplemental Table S3). Third, participants were school-age children from five mega-cities, which are more developed than other small cities and rural areas of China. Thus, our results cannot be generalized to children living in small cities or rural areas of China. Fourth, other factors may modify the associations between parental feeding practices and child weight status, such as child appetitive traits [49,50] and parenting style [51,52,53]. For example, maternal pressure to eat was associated with having a child with food avoidant tendencies (e.g., satiety responsiveness), which may modify the associations between pressure to eat and child weight status [54]. Parenting style modified the associations between parental feeding practices and weight status of children in Taiwan [8]. However, we did not assess these factors. Future studies are needed.

In conclusion, concern in feeding pattern was associated with increased risks of general and central obesity among both younger and older children, while pressure to eat was associated with reduced risks of general and central obesity only among younger children in mega-cities in China. Control was not associated with weight status among younger and older children. Child sex and maternal and paternal educations modified such associations. Future childhood obesity preventions may consider including strategies to assist parents to optimize parental feeding practices.

## Figures and Tables

**Table 1:** Description of factors of parental feeding practices arising from factor analysis based on pooled baseline data during 2015 to 2017 from the Childhood Obesity Study in China Mega-Cities (*n* = 2139).

Items	Factors		
	Concern	Pressure to Eat	Control
1. My child should always eat all the food on his/her plate.		0.77	
2. I have to make sure my child eats enough.		0.70	
3. I have to be sure my child eats during meal time.		0.52	
4. I will often encourage my child to eat healthy foods that they don’t like		0.46	
5. I offer snacks as a reward for good behavior.			0.75
6. I have to be sure my child does not eat too much.	0.61		
7. If I don’t regulate my child’s eating, he/she would eat less or more.			0.55
8. I am worried that my child will suffer from some diseases in the future due to poor diet, such as diabetes, heart disease.	0.76		
9. I am worried that my child will be overweight due to poor diet	0.79		
10. I allow my children to watch TV while eating.			0.61
11. I have to know when and what food my child eats every day.	0.53		
Eigenvalue	2.76	1.31	1.22
% Variance ^a^	25.10	11.90	11.10
Cronbach Alpha of “concern”, “pressure to eat”, and “control”	0.66	0.56	0.32
Mean±SD of all three subscales in all children	16.24 ± 2.25	16.00 ± 2.20	7.53 ± 1.95
Mean±SD of all three subscales in boys	16.29 ± 2.25	16.09 ± 2.26	7.53 ± 1.99
Mean±SD of all three subscales in girls	16.19 ± 2.25	15.90 ± 2.14	7.52 ± 1.92

Response of parental feeding practices measured scale using a 5-point Likert scale where “Strongly disagree” = 1 and “Strongly agree” =5. ^a^: Each factor’s variance contribution rate.

**Table 2:** Characteristics of children and their parents across parental feeding practices based on pooled baseline data during 2015 to 2017 from the Childhood Obesity Study in China Mega-Cities (*n* = 2139).

Characteristics	Concern ^a^			Pressure to Eat ^a^			Control ^a^		
	Tertile 1	Tertile 2	Tertile 3	Tertile 1	Tertile 2	Tertile 3	Tertile 1	Tertile 2	Tertile 3
Age (years)	11.30 ± 2.21	10.77 ± 2.16	10.83 ± 2.22	10.95 ± 2.23	10.92 ± 2.20	11.04 ± 2.19	10.90 ± 2.16	10.86 ± 2.24	11.14 ± 2.21
School type (%)									
Primary	46.09	57.91	58.28	53.68	56.20	52.38	55.90	57.36	49.00
Secondary	53.91	42.09	41.72 ***	46.32	43.80	47.62	44.10	42.64	51.00 **
BMI (kg/m^2^)	18.30 ± 3.23	18.55 ± 3.57	19.18 ± 3.91 ***	19.08 ± 3.61	18.63 ± 3.64	18.32 ± 3.50	18.52 ± 3.33	18.58 ± 3.72	18.93 ± 3.72 **
WHtR	0.42 ± 0.05	0.43 ± 0.05	0.44 ± 0.06 ***	0.44 ± 0.06	0.43 ± 0.05	0.43 ± 0.05	0.43 ± 0.05	0.43 ± 0.06	0.43 ± 0.06 ***
Central obesity (%) ^b^	12.71	18.74	23.47 ***	20.09	17.15	17.67	16.54	18.10	20.28
General obesity (%) ^c^	21.88	29.17	36.63 ***	34.67	27.47	25.50 ***	27.38	30.71	29.57
Maternal BMI (kg/m^2^)	22.00 ± 3.38	22.13 ± 3.39	22.11 ± 3.67	22.15 ± 3.85	22.08 ± 3.09	22.02 ± 3.48 ***	22.16 ± 4.19	21.89 ± 2.83	22.20 ± 3.29 ***
Paternal BMI (kg/m^2^)	23.79 ± 2.76	24.10 ± 2.91	24.05 ± 2.80	24.00 ± 2.78	24.05 ± 2.91	23.87 ± 2.78	23.95 ± 2.85	24.10 ± 2.87	23.87 ± 2.75
Maternal education (%)									
Middle school or lower	25.94	20.34	15.35	20.06	21.02	20.47	17.83	17.67	26.14
High or vocational school	30.03	27.80	26.05	27.80	27.51	28.53	27.60	28.84	27.40
College or above	44.03	51.86	58.60 ***	52.13	51.47	51.01	54.57	53.49	46.46 ***
Paternal education (%)									
Middle school or lower	20.97	14.20	12.23	16.30	14.88	16.19	14.22	14.98	18.23
High or vocational school	29.73	30.44	25.08	25.71	30.39	29.09	24.88	28.86	31.54
College or above	49.30	55.36	62.69 ***	57.99	54.73	54.72	60.90	56.16	50.24 **

BMI: Body mass index, WHtR: Waist to height ratio. Data were mean ± SD unless otherwise indicated. ^a^: The test of characteristics across tertiles for specific parental feeding practices were based on Chi-square test for categorical variables and ANOVA for continuous variables; ^b^: Central obesity was defined as having a WHtR ≥ 0.48; ^c^: General obesity was defined using sex-age-specific BMI cutoff points according to the Chinese National Standard, “WS/T 586-2018 Screening for overweight and obesity among school-aged children and adolescents”. In this study, general obesity included overweight and obesity. **: *p* < 0.01, ***: *p* < 0.001.

**Table 3:** Longitudinal associations between parental feeding practices and weight status of children, stratified by child age, child sex, and maternal and paternal educations—the Childhood Obesity Study in China Mega-Cities.

	BMI z-Score (Beta, 95% CI) ^a^		Waist-to-Height Ratio (Beta, 95% CI) ^a^	
	≤12 years (*n* = 1449)	>12 years (*n* = 692)	≤12 years (*n* = 1449)	>12 years (*n* = 692)
Among all				
Concern	0.13 (0.09, 0.17) ***	0.11 (0.07, 0.16) ***	0.01 (0.005, 0.01) ***	0.01 (0.004, 0.01) ***
Pressure to eat	−0.08 (−0.12, −0.04) ***	−0.04 (−0.09, 0.01)	−0.004 (−0.01, −0.001) **	−0.002 (−0.005, 0.001)
Control	0.01 (−0.03, 0.05)	0.03 (−0.02, 0.09)	0.001 (−0.002, 0.003)	0.002 (−0.001, 0.006)
Among boys				
Concern	0.16 (0.10, 0.23) ***	0.15 (0.08, 0.23) ***	0.01 (0.01, 0.02) ***	0.01 (0.005, 0.01) ***
Pressure to eat	−0.08 (−0.14, −0.02) *	0.01 (−0.09, 0.07)	−0.003 (−0.01, 0.001)	−0.0002 (−0.01, 0.005)
Control	0.04 (−0.02, 0.10)	0.05 (−0.03, 0.13)	0.002 (−0.002, 0.01)	0.005 (−0.0002, 0.01)
Among girls				
Concern	0.09 (0.04, 0.14) ***	0.08 (0.02, 0.14) *	0.01 (0.002, 0.01) ***	0.003 (−0.0001, 0.01)
Pressure to eat	−0.08 (−0.13, −0.03) **	−0.08 (−0.14, −0.01) *	−0.004 (−0.01, −0.001) *	−0.005 (−0.01, −0.001) *
Control	−0.03 (−0.08, 0.02)	0.02 (−0.05, 0.08)	−0.001 (−0.004, 0.002)	−0.0001 (−0.004, 0.004)
Children with maternal education lower than college				
Concern	0.15 (0.08, 0.22) ***	0.09 (0.03, 0.15) **	0.01 (0.005, 0.01) ***	0.01 (0.002, 0.01) **
Pressure to eat	−0.03 (−0.09, 0.03)	−0.04 (−0.11, 0.02)	0.001 (−0.003, 0.005)	−0.003 (−0.01, 0.001)
Control	−0.02 (−0.08, 0.04)	0.02 (−0.04, 0.09)	−0.002 (−0.01, 0.002)	0.002 (−0.002, 0.01)
Children with maternal education of college or above				
Concern	0.11 (0.06, 0.17) ***	0.12 (0.05, 0.19) ***	0.01 (0.004, 0.01) ***	0.01 (0.002, 0.01) **
Pressure to eat	−0.10 (−0.15, −0.05) ***	−0.06 (−0.13, 0.02)	−0.01 (−0.01, −0.003) ***	−0.003 (−0.01, 0.001)
Control	0.03 (−0.02, 0.08)	0.07 (−0.01, 0.14)	0.002 (−0.001, 0.01)	0.004 (−0.001, 0.01)
Children with paternal education lower than college				
Concern	0.13 (0.06, 0.20) ***	0.10 (0.03, 0.16) **	0.01 (0.004, 0.01) ***	0.01 (0.002, 0.01) **
Pressure to eat	−0.06 (−0.12, 0.01)	−0.03 (−0.11, 0.04)	−0.002 (−0.01, 0.002)	−0.002 (−0.01, 0.002)
Control	0.004 (−0.06, 0.07)	0.04 (−0.03, 0.11)	0.0004 (−0.004, 0.004)	0.003 (−0.001, 0.01)
Children with paternal education of college or above				
Concern	0.12 (0.07, 0.17) ***	0.13 (0.07, 0.20) ***	0.01 (0.005, 0.01) ***	0.01 (0.004, 0.01) ***
Pressure to eat	−0.09 (−0.14, −0.04) ***	−0.06 (−0.13, 0.01)	−0.01 (−0.01, −0.002) ***	−0.002 (−0.01, 0.002)
Control	0.03 (−0.02, 0.08)	0.04 (−0.03, 0.11)	0.001 (−0.002, 0.005)	0.002 (−0.002, 0.01)

Abbreviation: BMI: body mass index. ^a^: Child sex, child school level, child BMI z-score (or WHtR), child city of residence, maternal and paternal BMI, and maternal and paternal educations were adjusted as covariates in the mixed-effects model. In child age- and sex-stratified and parental education-stratified analyses, models were adjusted for the same variables except for child age and child sex and maternal and paternal educations. *: *p* < 0.05; **: *p* < 0.01; ***: *p* < 0.001. Numbers in bold indicated statistical significance.

## Data Availability

The datasets used and/or analyzed during the current study may be available from the corresponding author upon reasonable request.

## Supplementary Materials

The following are available online at https://www.mdpi.com/article/10.3390/nu14142797/s1, Supplemental Table S1: Characteristics of parental feeding practices and health outcomes by maternal and paternal educations, child sex and age—the Childhood Obesity Study in China Mega-Cities, Supplemental Table S2: Parental feeding practices and weight status indicators of children at baseline and follow-ups—the Childhood Obesity Study in China Mega-Cities, Supplemental Table S3: Longitudinal association between the three items of “Control” and child weight status, stratified by sex, maternal and paternal educations—the Childhood Obesity Study in China Mega-Cities (*n* = 2139), Supplemental Table S4: Family food environment across parental feeding practices tertiles based on pooled baseline data during 2015 to 2017 from the Childhood Obesity Study in China Mega-Cities (*n* = 2139).

## Abbreviations

WHtR: Waist-to-height ratio; BMI: Body mass index; CFQ: Child Feeding Questionnaire; ORs: Odds ratios; CI: Confidence interval.
