# Supplementary material for: A 3-Year Longitudinal Study of Effects of Parental Feeding Practices on Child Weight Status: The Childhood Obesity Study in China Mega-Cities
Source: Nutrients. 2022 Jul 7;14(14):2797. doi: 10.3390/nu14142797 (PMC9322165; doi:10.3390/nu14142797)
Supplement: Supplementary file 1 [file nutrients-14-02797-s001.zip › nutrients-1765461-supplementary.pdf]

Supplemental Table S1. Characteristics of parental feeding practices and health outcomes by maternal and paternal educations, child sex and age, the Childhood Obesity Study in China Mega-cities

	All (n=2,139)	Maternal education level		Paternal education level		Child sex		Child age groups	
		< college	≥ college [a]	< college	≥ college [a]	Boys	Girls [a]	≤ 12 years	> 12 years [a]
**Parental feeding practices**									
**Factor 1: Concern (%)** [b,f]									
1. I have to be sure my child does not eat too much.	1692 (82.10)	790 (79.48)	877 (85.15) ***	718 (79.25)	943 (84.95) ***	823 (81.73)	859 (82.52)	1186 (84.47)	506 (77.02) ***
2. I am worried that my child will suffer from some diseases in the future due to poor diet, such as diabetes, heart disease.	1884 (91.32)	900 (90.63)	957 (92.82)	827 (91.08)	1,019 (91.80)	921 (91.55)	952 (91.10)	1295 (92.37)	589 (89.11) *
3. I am worried that my child will be overweight due to poor diet	1825 (88.81)	851 (86.13)	946 (91.93) ***	777 (86.24)	1,012 (91.25) ***	890 (89.09)	924 (88.51)	1277 (91.54)	548 (83.03) ***
4. I have to know when and what food my child eats every day.	1680 (81.59)	783 (79.17)	871 (84.48) **	706 (78.10)	940 (84.68) ***	831 (82.85)	839 (80.36)	1185 (84.64)	495 (75.11) ***
**Factor 2: Pressure to eat (%)** [c,f]									
1. My child should always eat all the food on his/her plate.	1800 (86.79)	872 (87.11)	896 (86.57)	808 (88.31)	957 (85.91)	887 (87.73)	900 (85.71)	1237 (87.73)	563 (84.79)
2. I have to make sure my child eats enough.	1747 (84.52)	876 (87.69)	846 (81.98) ***	808 (88.50)	904 (81.51) ***	865 (85.90)	870 (83.09)	1186 (84.41)	561 (84.74)
3. I have to be sure my child eats during meal time.	1116 (54.23)	498 (50.15)	607 (59.05) ***	461 (50.72)	638 (57.74) ***	558 (55.58)	549 (52.74)	798 (56.84)	318 (48.62) **
4. I will often encourage my child to eat healthy foods that they don't like.	1926 (93.04)	919 (91.99)	976 (94.48) *	830 (91.01)	1,056 (94.96) ***	948 (93.95)	965 (92.08)	1340 (95.17)	586 (88.52) ***
**Factor 3: Control (%)** [b,f]									
1. I offer snacks as a reward for good behavior.	307 (14.85)	185 (18.54)	115 (11.13) ***	167 (18.31)	130 (11.70) ***	154 (15.28)	152 (14.53)	197 (14.00)	110 (16.67) *
2. I allow my children to watch TV while eating.	223 (10.89)	120 (12.20)	99 (9.66)	108 (12.03)	106 (9.60)	105 (10.58)	116 (11.12)	115 (8.24)	108 (16.56) ***
3. If I don't regulate my child's eating, he/she would eat less or more.	1126 (54.61)	541 (54.32)	567 (55.16)	495 (54.46)	603 (54.42)	558 (55.63)	560 (53.54)	807 (57.52)	319 (48.41) ***
**Health outcomes**									
1. BMI z-score	-0.05±0.89	0.01±0.88	-0.13±0.88 ***	0.05±0.89	-0.15±0.86 ***	0.09±0.92	-0.19±0.84 ***	-0.19±0.86	0.23±0.88 ***

2. Waist-to-height ratio	0.43±0.06	0.44±0.06	0.43±0.05	0.44±0.06	0.43±0.05 *	0.45±0.06	0.42±0.05 ***	0.44±0.06	0.43±0.06 **
3. General obesity (%) [d]	29.59	27.73	30.92	28.27	30.22	37.37	22.01 ***	32.15	24.24 ***
4. Central obesity (%) [e]	18.56	19.22	17.50	20.45	16.52 *	26.41	10.86 ***	19.28	17.05

Data are mean ± SD unless otherwise indicated.

a: *p*-value was based on rank sum test for categorical variables for parental feeding practices, t test for BMI *z*-score and waist-to-height ratio, and Chi-square test for prevalence of general obesity, and central obesity across maternal education, paternal education, child sex, and child age group.

b: Higher scores indicated worse parental feeding practices.

c: Higher scores indicated better parental feeding practices.

d: General obesity were defined using sex- and age-specific BMI cutoff points according to the Chinese National Standard, "WS/T 586-2018 Screening for overweight and obesity among school-aged children and adolescents". In this study, general obesity included both overweight and obesity.

e: Central obesity was defined as having a WHtR of 0.48 or higher.

f: In table, the frequencies were the total number of children agree and strongly agree on specific item of parental feeding parental practice, and proportions were the total number of agree and strongly agree divided by the total number of children reported on the item.

*: $p<0.05$; **: $p<0.01$; ***: $p<0.001$. Numbers in bold indicated statistical significance.

Supplemental Table S2. Parental feeding practices and weight status indicators of children at baseline and follow-ups, the Childhood Obesity Study in China Mega-cities

	Baseline	First follow-up	Second follow-up	Variability within individuals between first follow-up and baseline [a]	Variability within individuals between second and first follow-up [b]
Parental feeding practices					
Concern	16.24±2.25	16.22±2.38	16.13±2.28	-0.02±2.79	-0.02±2.77
Pressure to eat	16.00±2.20	16.08±2.27	16.01±2.16	0.08±2.70	0.05±2.63
Control	7.53±1.95	7.62±1.98	7.57±1.88	0.09±2.36	-0.05±2.22
BMI z-score	-0.15±0.83	0.05±0.86	0.08±0.89	0.21±0.40	0.22±0.28
Waist-to-height ratio	0.43±0.06	0.44±0.06	0.44±0.05	0.003±0.04	0.006±0.04
General obesity (%)	13.16	12.68	13.88	-0.48	1.20
Central obesity (%)	18.54	21.02	19.62	2.48	-1.40

Data were mean ± SD unless otherwise indicated.
a: Variability within individuals between first follow-up and baseline was calculated by data for first follow-up minus baseline;
b: Variability within individuals between second follow-up and first follow-up was calculated by data for second follow-up minus first follow-up.

Supplemental Table S3. Longitudinal associations between the three items of "Control" and child weight status indicators, stratified by child sex, maternal and paternal educations, the Childhood Obesity Study in China Mega-cities (n=2,139)

	BMI z-score (Beta, SE) [a]	Waist-to-height ratio (Beta, SE) [a]	General obesity (OR, 95%CI) [a]	Central obesity (OR, 95%CI) [a]
**All**				
1) I offer snacks as a reward for good behavior.	-0.004 (0.02)	-0.001 (0.001)	0.95 (0.81, 1.10)	1.03 (0.91, 1.15)
2) If I don't regulate my child's eating, he/she would eat less or more.	**0.05 (0.02)****	**0.004 (0.001)*****	**1.53 (1.23, 1.91)*****	**1.24 (1.10, 1.40)*****
3) I allow my children to watch TV while eating.	0.03 (0.02)	0.001 (0.001)	1.15 (0.99, 1.33)	1.07 (0.96, 1.18)
**Boys**				
1) I offer snacks as a reward for good behavior.	-0.01 (0.03)	-0.001 (0.002)	**0.71 (0.53, 0.95)***	0.99 (0.87, 1.12)
2) If I don't regulate my child's eating, he/she would eat less or more.	**0.08 (0.02)*****	**0.01 (0.002)*****	**1.74 (1.05, 2.89)***	**1.33 (1.18, 1.50)*****
3) I allow my children to watch TV while eating.	**0.06 (0.03)***	**0.003 (0.002)***	**3.19 (1.50, 6.78)****	**1.15 (1.005, 1.31)***
**Girls**				
1) I offer snacks as a reward for good behavior.	0.002 (0.02)	-0.00003 (0.001)	0.99 (0.85, 1.15)	1.22 (0.84, 1.78)
2) If I don't regulate my child's eating, he/she would eat less or more.	0.01 (0.02)	0.001 (0.001)	1.11 (0.97, 1.26)	1.17 (0.83, 1.65)
3) I allow my children to watch TV while eating.	0.003 (0.02)	-0.002 (0.001)	0.95 (0.82, 1.09)	0.91 (0.63, 1.32)
**Children with maternal education <college**				
1) I offer snacks as a reward for good behavior.	0.002 (0.02)	-0.0005 (0.001)	0.98 (0.87, 1.11)	0.98 (0.86, 1.12)
2) If I don't regulate my child's eating, he/she would eat less or more.	0.02 (0.02)	0.001 (0.001)	1.04 (0.92, 1.18)	**1.17 (1.02, 1.33)***
3) I allow my children to watch TV while eating.	0.008 (0.02)	-0.0001 (0.002)	0.99 (0.87, 1.13)	1.02 (0.89, 1.18)
**Children with maternal education ≥college**				
1) I offer snacks as a reward for good behavior.	-0.02 (0.02)	-0.001 (0.002)	0.91 (0.66, 1.26)	1.10 (0.94, 1.29)
2) If I don't regulate my child's eating, he/she would eat less or more.	**0.07 (0.02)****	**0.005 (0.001)*****	**2.08 (1.56, 2.79)*****	**1.32 (1.16, 1.50)*****
3) I allow my children to watch TV while eating.	**0.06 (0.02)***	0.002 (0.002)	**1.65 (1.24, 2.20)****	1.17 (0.98, 1.39)
**Children with paternal education <college**				
1) I offer snacks as a reward for good behavior.	0.01 (0.02)	0.001 (0.002)	0.58 (0.32, 1.04)	1.07 (0.86, 1.34)
2) If I don't regulate my child's eating, he/she would eat less or more.	0.01 (0.02)	0.001 (0.001)	1.29 (0.76, 2.17)	1.13 (0.97, 1.30)
3) I allow my children to watch TV while eating.	0.03 (0.02)	0.001 (0.002)	1.29 (0.96, 1.73)	1.07 (0.93, 1.24)
**Children with paternal education ≥college**				
1) I offer snacks as a reward for good behavior.	-0.01 (0.02)	-0.001 (0.001)	0.81 (0.59, 1.10)	1.08 (0.87, 1.34)
2) If I don't regulate my child's eating, he/she would eat less or more.	**0.07 (0.02)*****	**0.01 (0.001)*****	**1.88 (1.43, 2.48)*****	**1.60 (1.31, 1.95)*****
3) I allow my children to watch TV while eating.	**0.05 (0.02)***	0.001 (0.001)	**1.88 (1.38, 2.56)*****	1.19 (0.92, 1.55)

a: Child age, child sex, child school level, paternal and maternal BMI and educations were adjusted as covariates in the mixed-effects model. In sex-stratified and maternal and paternal educations-stratified analyses, models adjusted for the same variables except for child sex and maternal and paternal educations.
Variable definition: General obesity was defined using sex-age-specific BMI cutoff points according to the Chinese National Standard, “WS/T 586-2018 Screening for overweight and obesity among school-aged children and adolescents”. In this study, general obesity included both overweight and obesity. Central obesity was defined as having a waist to height ratio≥0.48.

Supplemental Table S4. Family food environment across parental feeding practices tertiles based on pooled baseline data during 2015 to 2017 from the Childhood Obesity Study in China Mega-cities (n=2,139)

Characteristics	Concern				Pressure to eat				Control			
	Tertile 1	Tertile 2	Tertile 3	*P* [a]	Tertile 1	Tertile 2	Tertile 3	*P* [a]	Tertile 1	Tertile 2	Tertile 3	*P* [a]
1. Frequency of buy and cook the following foods in the past month (%)												
1) Meat				0.193				**<0.001**				0.232
Never/ Rarely	17.40	16.30	16.46		19.84	18.04	12.29		16.02	17.52	16.61	
Often	33.23	40.53	34.63		36.88	38.41	33.13		34.21	37.83	36.36	
Every day	49.37	43.17	48.91		43.28	43.55	54.59		49.77	44.65	47.02	
2) Egg				0.06				**<0.01**				**<0.01**
Never/ Rarely	13.99	12.11	9.47		14.69	11.23	9.64		10.71	10.42	14.44	
Often	37.89	38.66	37.27		38.28	39.63	35.93		34.16	39.50	40.19	
Every day	48.11	49.22	53.26		47.03	49.14	54.43		55.12	50.08	45.37	
3) Milk				**0.01**				**<0.01**				**<0.001**
Never/ Rarely	19.53	14.88	14.69		17.68	16.22	15.16		13.13	16.85	19.09	
Often	26.61	25.58	24.69		28.48	26.52	21.88		23.13	25.43	28.33	
Every day	53.86	59.53	60.63		53.83	57.25	62.97		63.75	57.72	52.58	
4) Vegetables				**<0.001**				**0.03**				**<0.001**
Never/ Rarely	2.81	1.87	2.17		3.74	1.56	1.55		2.17	1.86	2.82	
Often	17.50	12.75	8.98		13.73	14.49	10.99		9.75	12.87	16.61	

Every day	79.69	85.38	88.85		82.53	83.96	87.46		88.08	85.27	80.56	
5) Fruit				**<0.01**				0.139				**0.02**
Never/Rarely	4.84	3.73	5.12		4.84	4.51	4.33		3.25	4.50	5.95	
Often	25.27	19.88	16.15		21.56	21.93	17.80		18.11	21.58	21.60	
Every day	69.89	76.40	78.73		73.59	73.56	77.86		78.64	73.91	72.46	
2. Parental modeling with unhealthy eating habits				**<0.001**				**0.001**				**<0.001**
1) Frequency of eating out of parents	2.03±2.42	1.96±2.05	2.05±2.23		2.18±2.37	1.95±2.05	1.90±2.27		1.93±2.08	1.96±2.14	2.15±2.47	

Data were mean ± SD unless otherwise indicated.
a: Chi-square test was used to examine difference of family food environment across parental feeding practices tertiles.
